# Efficacy and Safety of Advanced Endoscopic Techniques in Early Barrett’s Neoplasia: A Systematic Review and Pooled Analysis

**DOI:** 10.7759/cureus.86015

**Published:** 2025-06-14

**Authors:** Neelam Khetpal, Saeed Ali, Sana Hussain, Asad Ali, Mohamad Sharbatji, Christopher Childs, Muhammad Ali Khan, Muhammad K Hasan

**Affiliations:** 1 Gastroenterology and Hepatology, Cleveland Clinic Florida, Weston, USA; 2 Internal Medicine, AdventHealth/Florida Hospital, Orlando, USA; 3 Gastroenterology and Hepatology, SUNY Upstate Medical University, Syracuse, USA; 4 Hardin Library for the Health Sciences, University of Iowa, Iowa City, USA; 5 Gastroenterology and Hepatology, University of Texas MD Anderson Cancer Center, Houston, USA; 6 Gastroenterology, Center for Interventional Endoscopy, AdventHealth Orlando, Orlando, USA

**Keywords:** barrett’s esophagus-related high-grade dysplasia, early adenocarcinoma, endoscopic submucosal dissection, focal endoscopic mucosal resection followed by radiofrequency ablation, stepwise/complete emr

## Abstract

Focal endoscopic mucosal resection (f-EMR) followed by radiofrequency ablation (f-EMR+RFA), stepwise/complete EMR (c-EMR), and endoscopic submucosal dissection (ESD) are used to manage Barrett’s esophagus (BE)-related high-grade dysplasia (HGD) and early adenocarcinoma (EAC). We present a systematic review and meta-analysis evaluating these modalities' cumulative and comparative efficacy and safety. We evaluated studies reporting efficacy and safety of ESD, f-EMR+RFA, and c-EMR for BE-related early neoplasia management. Primary outcomes were recurrence of HGD or EAC and risk of strictures, perforation, and bleeding. Secondary outcomes were en bloc and R0 resections for ESD and complete eradication of neoplasia for f-EMR-RFA and c-EMR.

Thirty-eight studies with 2,434 patients (684 ESD, 938 f-EMR+RFA, 812 c-EMR) were included. Weighted pooled rates (WPR) for recurrence were 10.3% (ESD), 5% (f-EMR+RFA), and 7.4% (c-EMR). There was no difference in recurrence with any endoscopic modality (p>0.05). WPR for strictures was 9.5% (ESD), 11.5% (f-EMR+RFA), and 29% (c-EMR). ESD and f-EMR+RFA were associated with lower stricture formation compared to c-EMR (p<0.05), while there was no difference seen between ESD and f-EMR+RFA. WPR for perforation was 3.7% (ESD), 1.6% (f-EMR+RFA), and 2% (c-EMR). F-EMR+RFA was associated with a lower risk of perforation compared to ESD (p=0.01), while no difference was found between ESD and c-EMR. WPR for bleeding was 3.5% (ESD), 3% (f-EMR+RFA), and 6% (c-EMR). There was no difference in the recurrence of neoplasia with any endoscopic modality. f-EMR+RFA appears to be the preferred endoscopic modality for the management of BE-related neoplasia.

## Introduction and background

Barrett’s esophagus (BE) can develop in patients with long-standing gastroesophageal reflux disease (GERD). It is defined as replacing the normal squamous epithelium of the lower esophagus with a columnar epithelium containing specialized intestinal metaplasia (IM) [1]. BE is a significant risk factor for the development of adenocarcinoma of the esophagus (EAC), with an increased annual risk of 0.25% for patients without dysplasia and 6% in patients with high-grade dysplasia (HGD) [2]. The incidence of esophageal adenocarcinoma has increased six-fold over the last three decades along with a seven-fold increase in mortality [3].

Endoscopic mucosal resection (EMR) has become the standard of care for the management of BE-related HGD or EAC [1,4]. However, if adenocarcinoma has invaded the submucosa, esophagectomy is recommended. Due to the risk of metachronous lesions in residual BE, complete eradication is recommended [1,5]. This can be achieved by complete or stepwise EMR (c-EMR), which is an extensive EMR of BE-related neoplasia with the intent to cure. Another strategy for the management of BE-related neoplasia is to use a multistep approach with focal EMR followed by successive radiofrequency ablation (f-EMR+RFA) therapy sessions.

Endoscopic submucosal dissection (ESD) is an advanced and relatively newer technique that has been utilized in the management of early cancers of the GI tract. In comparison to EMR, ESD has the advantage of providing en bloc resections irrespective of lesion size. It has been utilized well in Asia for the management of squamous cell neoplasia of the esophagus [6]. Theoretically, because of en bloc curative resections, ESD should provide lower recurrence rates compared to conventional EMR. To date, there has been only one randomized controlled trial (RCT) comparing ESD with EMR for the management of BE-related neoplasia, which did not show any benefit in recurrence despite ESD being associated with higher en bloc resection rates [7].

This systematic review and pooled analysis assessed the overall efficacy and safety of f-EMR+RFA, c-EMR, and ESD in managing BE-related neoplasia. Due to a lack of studies comparing their effectiveness and safety, we also conducted an indirect comparison analysis to evaluate the performance of all three endoscopic modalities.

## Review

Methods

Identification and Retrieval of Primary Studies

We followed the guidelines of Preferred Reporting Items for Systematic Reviews and Meta-Analyses (PRISMA) and meta-analysis of observational studies in epidemiology (MOOSE) to conduct this systematic review and meta-analysis [8,9]. An experienced medical reference librarian (CC) performed the search strategy and subsequent literature search. The search strategies were developed in Ovid MEDLINE and translated to match the subject headings and keywords for Ovid Embase, Cochrane database, ISI Web of Science, and Scopus from inception through April 20, 2020. The following MeSH, Emtree, and keyword search terms were used: “esophagus” OR “esophageal,” “Barrett” OR “Barrett’s,” “esophageal adenocarcinoma” OR “esophageal cancer” AND “resection,” OR “endoscopic resection” OR “endoscopic mucosal resection” OR “EMR” OR “ablation” OR “radiofrequency ablation” OR “RFA” OR “submucosal dissection” OR “endoscopic submucosal dissection” OR “ESD.” The search accounted for plurals and variations in spelling with the use of appropriate wildcards. Selection of articles for full-text review was based on an initial assessment of titles and abstracts. To maximize the comprehensiveness of the search, a manual review of the reference lists of the retrieved publications (i.e., backward snowballing) was undertaken. All identified records were imported into EndNote version 9.0 (Philadelphia, PA: Thomson ISI ResearchSoft), a reference management software, and duplicate citations were systematically removed.

Inclusion and Exclusion Criteria

Eligibility criteria were determined a priori by two study authors (MK and MA) and included patients aged ≥18 years with Barrett’s esophagus and HGD or EAC (intramucosal carcinoma limited to the mucosa, stage T1a) undergoing treatment with one of the following: f-EMR+RFA, c-EMR, or ESD with intent of complete eradication of Barrett’s esophagus-related neoplasia. Our predetermined inclusion criteria were observational studies and randomized controlled trials (RCTs) that reported clinical outcomes, including complete eradication rates of HGD or EAC, en bloc resection rates, curative resection rates, recurrence rates, and adverse events. To minimize bias inherently associated with individual case reports and small case series, we only included studies with 10 or more patients and follow-up of at least one year after complete eradication (to assess the effect of therapy and evaluate for recurrence), which was the most important comparative factor for all three modalities. For f-EMR+RFA studies, we only included studies in which more than 70% of patients underwent f-EMR; this cutoff was selected for estimating the effect of f-EMR with additional RFA in a pooled analysis of the f-EMR+RFA group. Studies were excluded based on the following criteria: case report/series with <10 patients, review articles, commentaries, animal studies, ESD for EAC not arising from BE, studies not reporting biopsy results, and when the aforementioned variables of interest were not reported. If there was any suspicion of cohort overlap between studies, we included only the most recent study. Only studies published in the English language and in peer-reviewed journals were included in the analysis. Published abstracts or unpublished data were excluded, as there is a discrepancy between full publications and unpublished data or published abstracts [10,11].

Study Selection and Data Extraction

Two reviewers (SA and NK) independently evaluated the eligibility of the identified studies, extracted data using standardized data extraction forms, and assessed the methodological quality of each included study. Discrepancies between reviewers were to be resolved through discussion with a third reviewer (MK), with consensus serving as the final decision-making approach. Extracted data included study design, country, year of publication, patient demographics, BE length, histopathology (presence or absence of HGD or EAC), mean/median follow-up duration, adverse events (specifically, bleeding), stricture formation, and perforations. Upon completion of data extraction, the data sheets were compared, and any discrepancies between reviewers were addressed through consultation with a third reviewer (MK), with consensus used to resolve all disagreements.

Definitions

Barrett’s esophagus-related neoplasia was defined as histologically confirmed Barrett’s mucosa with high-grade dysplasia or superficial early adenocarcinoma limited to mucosa not exceeding stage T1a. Recurrence was defined as the presence of histologically confirmed BE neoplasia at the index resection site on subsequent endoscopies during the follow-up period.

For ESD, en bloc resection was defined as a complete excision of the target lesion that was then retrieved as a single specimen. R0 resection was defined as a specimen with negative lateral and deep margins for BE dysplasia/or EAC. Curative resection was defined when all of the following were present: R0 resection, well to moderately differentiated histology, and absence of lymphovascular invasion.

Data Synthesis and Statistical Analysis

Our primary outcome of interest was the rate of recurrence after complete eradication with one of the treatment modalities. Weighted pooled rates (WPR) along with 95% confidence intervals were calculated for primary outcomes of interest, and these were analyzed using the DerSimonian-Laird random effects model of meta-analysis [12]. For patients undergoing either f-EMR+RFA or c-EMR, we also calculated WPR for eradication rates of BE-associated neoplasia. For patients undergoing ESD, WPRs were calculated for en bloc resections, R0, and curative resection rates. For all patients, WPR was also calculated for the following three main adverse events: bleeding, stricture formation, and perforation.

As secondary outcomes, we also indirectly compared each treatment modality with the other. We calculated the proportional difference of WPR in terms of rates of recurrence, stricture formation, bleeding, and perforation. Heterogeneity among the included studies was evaluated using Cochran’s Q test and the I² statistic. A p-value less than 0.1 on Cochran’s Q test was considered indicative of statistically significant heterogeneity. I² values were interpreted as follows: 0-40% as low, 30-60% as moderate, 50-90% as substantial, and 75-100% as considerable heterogeneity.

Quality Assessment of Studies

Two reviewers (SA and AA) independently assessed the quality of studies, and any disagreement between reviewers was resolved with consensus. Quality assessment of observational studies was done using the National Institutes of Health (NIH) quality assessment tool for before-after studies with no control group. The Cochrane tool for assessing the risk of bias was used for evaluating the quality of RCTs.

Results

Search Strategy Yield, Study Characteristics, and Quality Assessment

**Figure 1:**
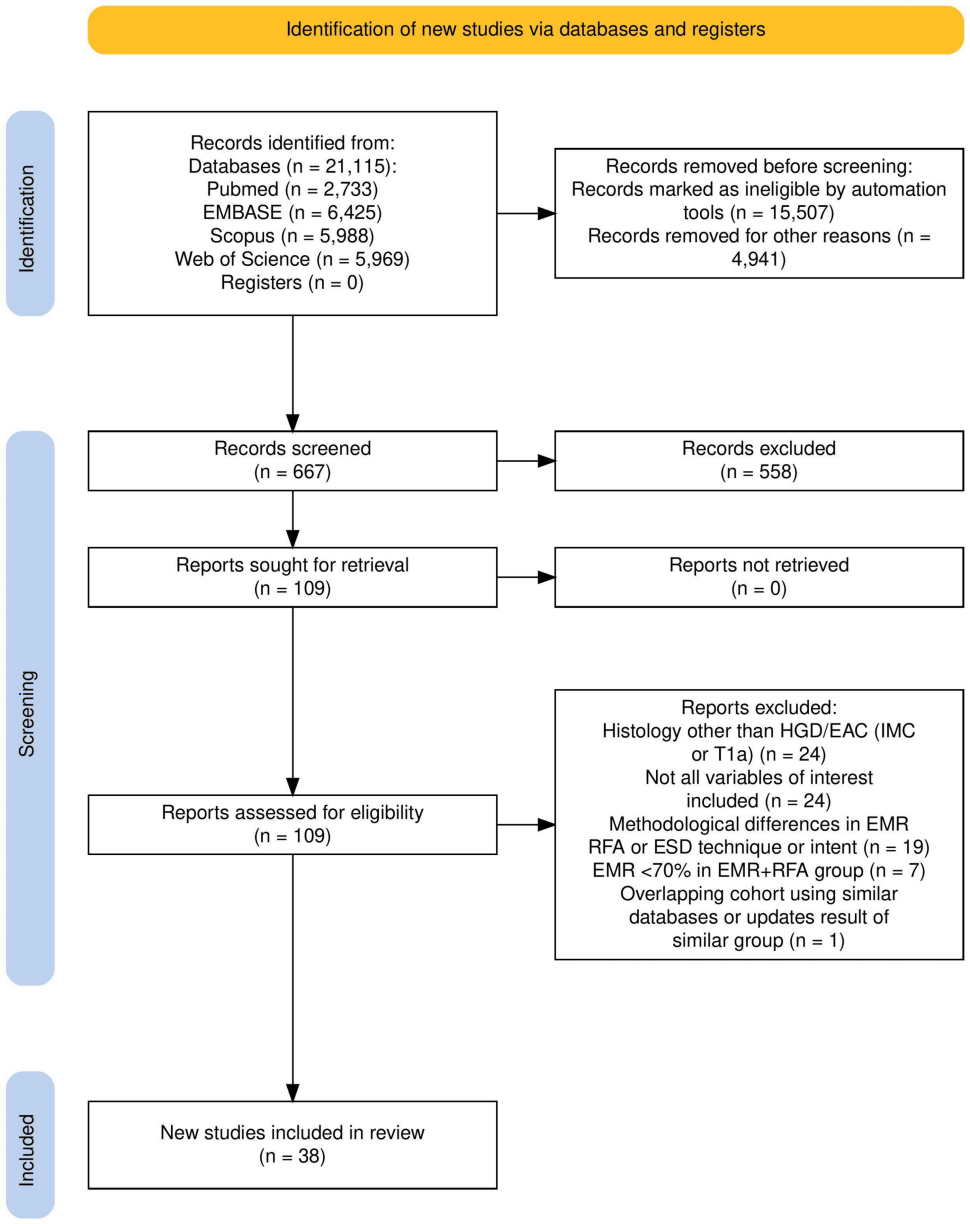
PRISMA flow diagram summarizing the identification and selection process of the studies. HGD: high-grade dysplasia; EAC: early adenocarcinoma; IMC: intra-mucosal carcinoma; ESD: endoscopic submucosal dissection; f-EMR+RFA: focal endoscopic mucosal resection + radiofrequency ablation; c-EMR: complete endoscopic mucosal resection PRISMA: Preferred Reporting Items for Systematic Reviews and Meta-Analyses

The search strategy identified 21,115 studies, of which 15,507 were excluded as duplicates (Figure 1). Of the remaining 5,608 studies, 4,941 were excluded after title and abstract review. A bibliographic review of 667 articles did not reveal any additional studies meeting our inclusion criteria. Therefore, a total of 38 studies were included in this systematic review and meta-analysis [1,4,7,13-47]. Of these, two studies were RCTs, while the remaining were observational studies. One RCT compared ESD with f-EMR+RFA [7], and the other compared f-EMR+RFA with c-EMR [1]. One observational study by Genere et al. compared ESD with c-EMR [47]. Therefore, 15 studies included patients undergoing ESD [4,7,35-47], 14 studies included patients undergoing f-EMR+RFA [1,7,13-22,24,28], and 12 studies included patients undergoing c-EMR [1,23,25-27,29-34,47]. Among the f-EMR+RFA studies, one cohort study contained an overlapping patient population from four other studies and presented data for the recurrence and eradication of BE neoplasia only [1,16,21,22,28]. Therefore, for the calculation of recurrence rates and eradication of BE neoplasia, we only included the study by Phoa et al., while for the calculation of adverse events rates, we included the aforementioned four studies individually [16].

A total of 2,434 patients were included in our analysis, 938 who underwent f-EMR+RFA, 812 who underwent c-EMR, and 684 who underwent ESD. Among the patients undergoing ESD, 83% were men with ages ranging from 60 to 73 years. Likewise, 79% and 86% were men with ages ranging between 60-68 years and 64-68 years among patients undergoing f-EMR+RFA and c-EMR, respectively. Details of the study and patient characteristics are outlined in Tables 1-3.

**Table 1:** Characteristics of studies and patients included in f-EMR+RFA studies. HGD: high-grade dysplasia; EAC: early adenocarcinoma; EMR: endoscopic mucosal resection; BE: Barrett’s esophagus; f-EMR+RFA: focal endoscopic mucosal resection + radiofrequency ablation; LGD: low-grade dysplasia

Studies	Study type	Country	No. centers	No. patients	Mean age (years)	Males (n)	HGD (n)	EAC (n)	BE length (cm) mean (SD) or median (IQR)	Follow-up (months), mean (SD) or median (range)
Ganz et al. (2008) [13]	Retrospective	USA	16	24 (subgroup analysis)	66	NA	19	5	6 (3-8)	12
Okoro et al. (2012) [14]	Retrospective	USA	1	44	66	41	35	4	6.9 (0.5)	20.5
Caillol et al. (2012) [15]	Retrospective	France	1	16	60	14	13	0	2 (3-5)	15
Phoa et al. (2013) [16]	Prospective	Europe	4	55	65	45	14	23	5 (4-8)	61
Saligram et al. (2013) [17]	Retrospective	USA	1	54	68	45	0	54	4.5 (3.9)	23
Phoa et al. (2016) [18]	Prospective	Europe	13	132	65	107	31	76	6 (4-9)	27
Li et al. (2016)[19]	Prospective	USA	148	406	67	350	252	154	4.6 (3.6)	34.3 (18.4)
Agoston et al. (2016) [20]	Retrospective	USA	4	78	67	59	0	78	4.1 (3.7)	26.4 (2-116)
Barret et al. (2016) [24]	Retrospective	Netherlands	2	40	66	31	11	27	2 (2-4)	19 (6-40)
van Vilsteren et al. (2011) [1]	Randomized	Netherlands, Germany	3	22	Median 69 (55-73)	19	7	15	4 (2-5)	22 (IQR 17-30)
Gondrie et al. (2008) [21]	Prospective	Netherlands	1	11	Median 60 (57-67)	8	HGD 9; LGD 2	0	5 (4-7)	19 (18-22)
Gondrie et al. (2008) [22]	Prospective	Netherlands	1	12	Median 70 (53-76)	9	HGD 11; LGD 1	0	7 (6.5-8)	14
Pouw et al. (2010) [28]	Prospective cohort	Netherlands, Germany, Belgium	3	24	65	20	7	16	8	22 (17.2-23.8)

**Table 2:** Characteristics of studies and patients included in c-EMR groups. HGD: high-grade dysplasia; EAC: early adenocarcinoma; c-EMR: complete endoscopic mucosal resection; BE: Barrett’s esophagus

Studies	Study type	Country	No. centers	No. patients	Mean age (years)	Males (n)	HGD (n)	EAC (n)	BE length (cm), mean (SD) or median (IQR)	Median follow-up, months
Larghi et al. (2007) [25]	Prospective	USA	2	24	64.1	18	19	5	2.5 (1-8)	28
Lopes et al. (2007) [26]	Retrospective	France	1	41	65.8	35	18	23	4.9 (3.4)	31.6
Bhat et al. (2009) [27]	Retrospective	USA	1	60	67	53	60	26	4.2 (3.4)	15
Pouw et al. (2010) [23]	Retrospective	Europe	4	169	64	151	70	67	3 (2-5)	32
Brahmania et al. (2010) [29]	Retrospective	Canada	1	22	67	22	15	3	5.5 (3.5)	24
van Vilsteren et al. (2011) [1]	Prospective	Europe	3	25	68	21	12	13	4 (2-5)	24
Gerke et al. (2011) [30]	Retrospective	USA	1	41	67	32	26	11	3.2 (2.3)	25.4
Chung et al. (2011) [31]	Retrospective	Australia	2	77	65	64	69	8	2 (1-3)	17
Conio et al. (2014) [32]	Retrospective	Italy	1	47	65	43	37	10	3 (3-12)	18.4
Konda et al. (2014) [33]	Retrospective	USA	1	107	67.5	78	63	39	2.5 (2-5)	40.6
Bahin et al. (2016) [34]	Prospective	Australia	2	138	66	128	127	26	2.7 (1-4)	54.7

**Table 3:** Characteristics of studies and patients included in ESD groups. HGD: high-grade dysplasia; EAC: early adenocarcinoma; BE: Barrett’s esophagus; ESD: endoscopic submucosal dissection

Studies	Study type	Country	No. patients	No. lesions	Age (years)	Males (n)	HGD (n)	EAC (n)	BE lesion size (mm)	Follow-up
Neuhaus et al. (2012) [35]	Prospective	Germany	30	30	Median 60 (range: 29-86)	21	2	24	Median 20 (range: 10-30)	Median 17 months
Hoteya et al. (2013) [36]	Retrospective	Japan	25	25	Mean 63.5+12.5	22	0	25	Mean 20.2±17.6	Median 34 months (range: 2-96)
Nagami et al. (2014) [37]	Retrospective	Japan	14	14	Mean 61.4-14.2	13	0	14	Median 18 (range: 8-30)	(1.6-87.6 months)
Kagemoto et al. (2014) [38]	Retrospective	Japan	23	26	Mean 60+10	21	0	26	Mean 19±13.6	33±24 months
Probst et ai. (2015) [39]	Prospective	Germany	87	87	Mean 66.2+9.6	71	0	88	Mean 21 (range: 10-50)	24.3 months (range: 3-70 months)
Chevaux et al. (2015) [40]	Retrospective	Belgium	75	75	Median 68 (IQR: 61-76)	63	11	55	Median 20 (IQR: 10-30)	20
Höbel et al. (2015) [41]	Retrospective	Germany	22	22	Mean 64.1+19.7	20	0	20	Mean 44 (range: 18-120)	1.6 years (range: 1 month-4.5 years)
Barret et al. (2016) [42]	Retrospective	France	35	36	Mean 66.2+12	29	6	29	Mean 50.6±22	Mean 12.9±9 months
Terheggen et al. (2017) [7]	Randomized	Germany	20	20	Mean 64+12	NA	0	17	Mean 16±7	23.1
Yang et al. 2017 [43]	Retrospective	USA	46	46	Median 69 (range: 42-82)	39	14	32	Median 45 (range: 13-125)	11.3
Subramaniam et al. (2017) [4]	Retrospective	Europe	124	143	Mean 71.2	97	18	113	Mean 31.1 (range: 5-90)	21.6
Shimizu et al. (2018) [44]	Retrospective	Japan	86	91	63.6 (10.8)	77	0	91	Mean 23.2±3.0	Median 28.5 months
Probst et al. (2019) [45]	Retrospective	Germany	23	23	Median 67 (45-84)	21	0	23	Median 40 (range: 20-60)	Median 21 months (range: 3-54).
Podboy et al. 2020 [46]	Retrospective	USA	20	20	Mean 70.9	18	6	10	Mean 13.9+5.5	1.4±1.1 years
Genere et al. (2020) [47]	Retrospective	USA	54	72	Mean 71	52	15	30	Mean 21	NA

Both RCTs (van Vilsteren et al. and Terheggen et al.) had a high risk of performance bias as neither the patients nor the endoscopists could be blinded to the type of procedure [1,7]. These trials also had an unclear risk of detection bias. There was a low risk of selection, attrition, and reporting biases in both RCTs. We used the NIH assessment tool for before-after studies with no control group for quality assessment of observational studies. Twenty-eight studies were rated as high quality, while the remaining eight were of fair quality.

Efficacy and Safety of ESD

A total of 15 studies reported efficacy and safety outcomes with ESD [4,7,35-47]. En bloc resection was defined as the resection of the targeted lesion as one specimen. WPR with 95% CI for en bloc resection was 91% (88%, 93%), Cochran’s Q test: p=0.13, I²=32%. WPR with 95% CI for R0 was 81% (77%, 84%), Cochran’s Q test: p=0.36, I²=8%. WPR for curative resection was 69% (60%, 77%), Cochran’s Q test: p <0.01, I²=71%.

WPR for recurrence of BE-related neoplasia during follow-up was 9.5% (7%, 13%), Cochran’s Q test: p=0.52, I²=0%. WPR for risk of bleeding requiring further intervention or hospitalization was 3% (2%, 5%), Cochran’s Q test: p=0.68, I²=0%, WPR for risk of developing strictures during follow-up was 9.6% (4%, 21%), Cochran’s Q test: p<0.01, I²=90%. Chevaux et al. reported a 60% stricture development rate, which was an outlier compared to other studies [40]. On sensitivity analysis after excluding this outlier, WPR was 8% (5%, 14%), Cochran’s Q test: p=0.11, I²=48%. WPR for risk of perforation was 4% (2%, 6%), Cochran’s Q test: p=0.77, I²=0%.

Efficacy and Safety of f-EMR+RFA

Fourteen studies were included in the analyses for f-EMR+RFA [1,7,13-22,24,28]. WPR for complete eradication of BE-related neoplasia was 92% (88%, 94%), Cochran’s Q test: p=0.15, I^2^=33%. WPR for recurrence of BE-related neoplasia during follow-up was 5% (2%, 10%), Cochran’s Q test: p=0.004, I²=63%. Okoro et al. reported a significantly higher recurrence rate (21%) compared to other studies [14]. On sensitivity analysis, WPR for recurrence was 4% (2.5%, 7%), Cochran’s Q test: p=0.17, I²=15%. WPR for risk of bleeding was 3% (2%, 5%), Cochran’s Q test: p=0.15, I²=28%, WPR for risk of strictures during follow-up was 11.5% (7%, 17%), Cochran’s Q test: p<0.001, I²=71% and WPR for risk of perforation was 1.6% (0.8%, 3%), Cochran’s Q test: p=0.57, I²=0%.

Efficacy and Safety of c-EMR

Twelve studies evaluated patients undergoing c-EMR [1,23,25-27,29-34,47]. WPR for complete eradication of BE-related neoplasia was 94% (88%, 97%), Cochran’s Q test: p<0.001, I²=74%. WPR for risk of recurrence of BE-related neoplasia was 6.6% (4%, 10%), Cochran’s Q test: p=0.10, I²=47%. WPR for risk of bleeding was 6% (4%, 10%), Cochran’s Q test: p=0.05, I²=52%. WPR for stricture formation during follow-up was 29% (21%, 39%), Cochran’s Q test: p<0.001, I²= 82%, and WPR for risk of perforation was 2% (1%, 4%), Cochran’s Q test: p=0.86, I²=0%.

Indirect Comparison Analyses for all Three Modalities

**Figure 2:**
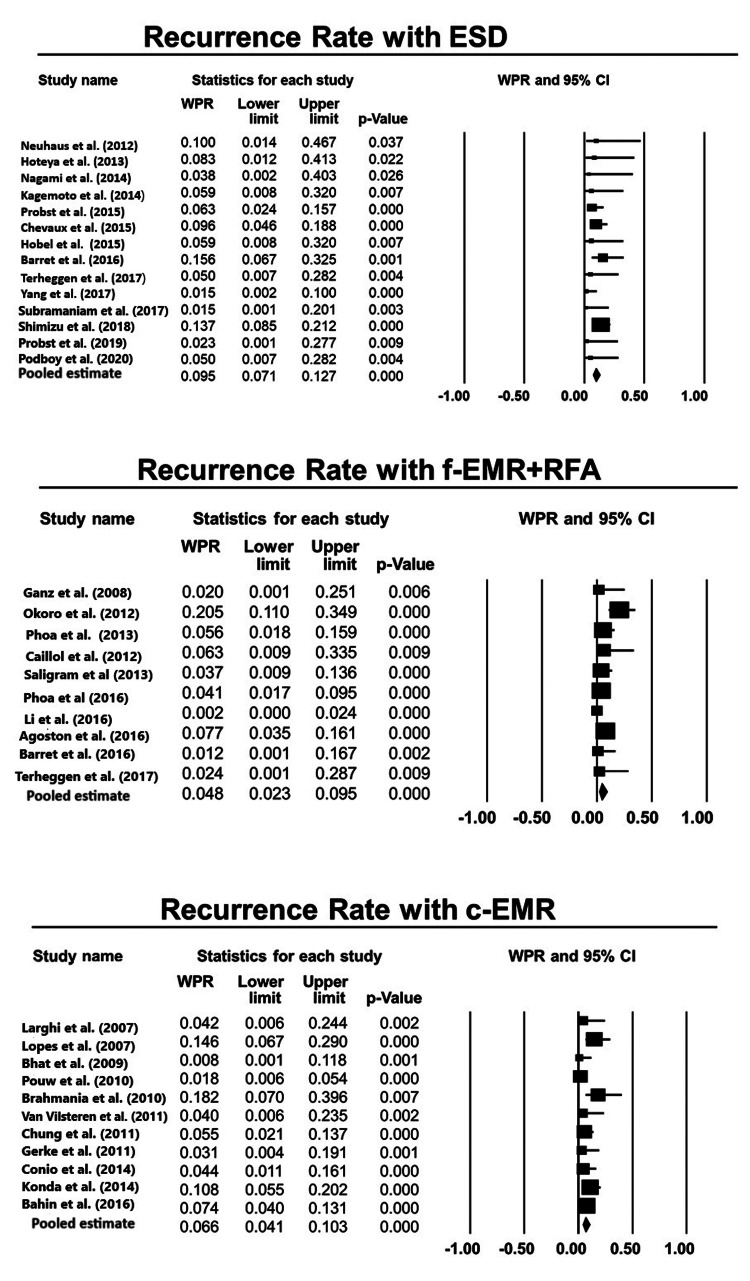
Forest plots for the included studies evaluating the rate of recurrence for each technique, viz., EMR, ESD, and RFA. ESD: endoscopic submucosal dissection; f-EMR+RFA: focal endoscopic mucosal resection + radiofrequency ablation; c-EMR: complete endoscopic mucosal resection Weighted pooled rates (WPR) along with 95% confidence intervals (CIs)

Rate of recurrence: The proportional difference of WPR with 95% CI for risk of recurrence of BE-related neoplasia while comparing ESD with f-EMR +RFA was 2% (-1%, 5%), p=0.22 (Figure 2) [1,4,7,13-20,23-27,29-46]. The proportional difference while comparing ESD with c-EMR was 1.5% (-2%, 5%), p=0.39, and the proportional difference while comparing f-EMR+RFA and c-EMR was 0.5% (-2%, 3%), p=0.73. Therefore, there was no difference in all three modalities in terms of risk of recurrence of BE-related neoplasia (Table 4).

**Table 4:** Proportional difference for comparative analysis of ESD, f-EMR+RFA, and c-EMR. f-EMR+RFA: focal endoscopic mucosal resection + radiofrequency ablation; ESD: endoscopic submucosal dissection; c-EMR: complete endoscopic mucosal resection Weighted pooled rates (WPR) along with 95% confidence intervals (CIs).

Outcome	ESD vs f-EMR+RFA	ESD vs c-EMR	f-EMR+RFA vs c-EMR
Recurrence of neoplasia	2% (-1%, 5%), p=0.22	1.5% (-2%, 5%), p=0.39	0.5% (-2%, 3%), p=0.73
Strictures	2% (-1.0%, 5%), p=0.41	19% (15%, 23%), p<0.001 in favor of ESD	17% (13%, 21%), p<0.001 in favor of f-EMR+RFA
Perforations	2% (0.5%, 3%), p=0.01 in favor of f-EMR+RFA	1% (-0.1%, 3%), p=0.07	-0.4% (-1%, 1%), p=0.05
Bleeding	0.5% (-1%, 2%), p=0.58	3% (1%, 5%), p=0.01 in favor of ESD	3.6% (1.5%, 6%), p=0.001 in favor of f-EMR+RFA

**Figure 3:**
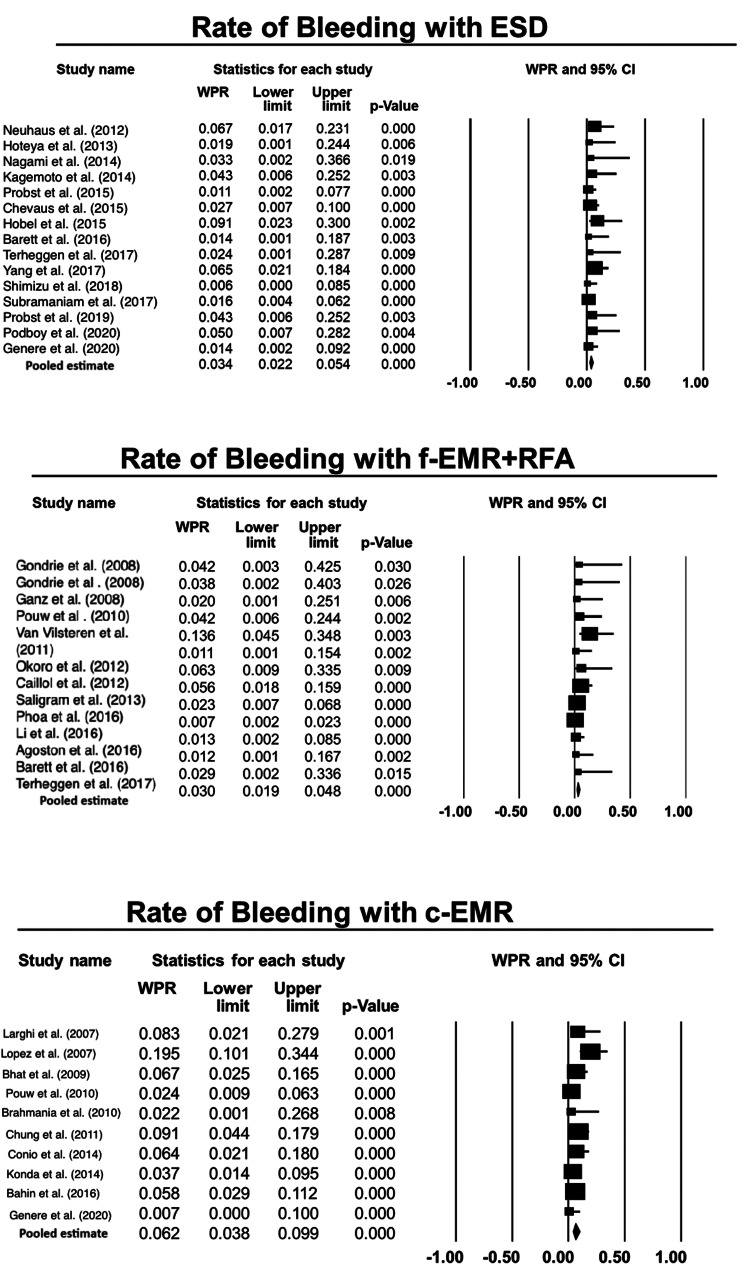
Forest plots for the included studies evaluating the rate of bleeding associated with each technique, viz., EMR, ESD, and RFA. ESD: endoscopic submucosal dissection; f-EMR+RFA: focal endoscopic mucosal resection + radiofrequency ablation; c-EMR: complete endoscopic mucosal resection Weighted pooled rates (WPR) along with 95% confidence intervals (CIs).

Risk of bleeding: The proportional difference for risk of bleeding while comparing ESD with f-EMR+RFA was 0.5% (-1%, 2%), p=0.58 (Figure 3) [1,4,7,13-15,17-27,29,31-47]. The proportional difference comparing ESD with c-EMR was 3% (1%, 5%), p=0.01 in favor of ESD. Finally, the proportional difference comparing f-EMR+RFA and c-EMR was 3.6% (1.5%, 6%), p=0.001 in favor of f-EMR+RFA. Therefore, ESD and f-EMR+RFA were associated with significantly less bleeding than c-EMR. However, there was no difference in terms of bleeding while comparing ESD with f-EMR+RFA (Table 4).

**Figure 4:**
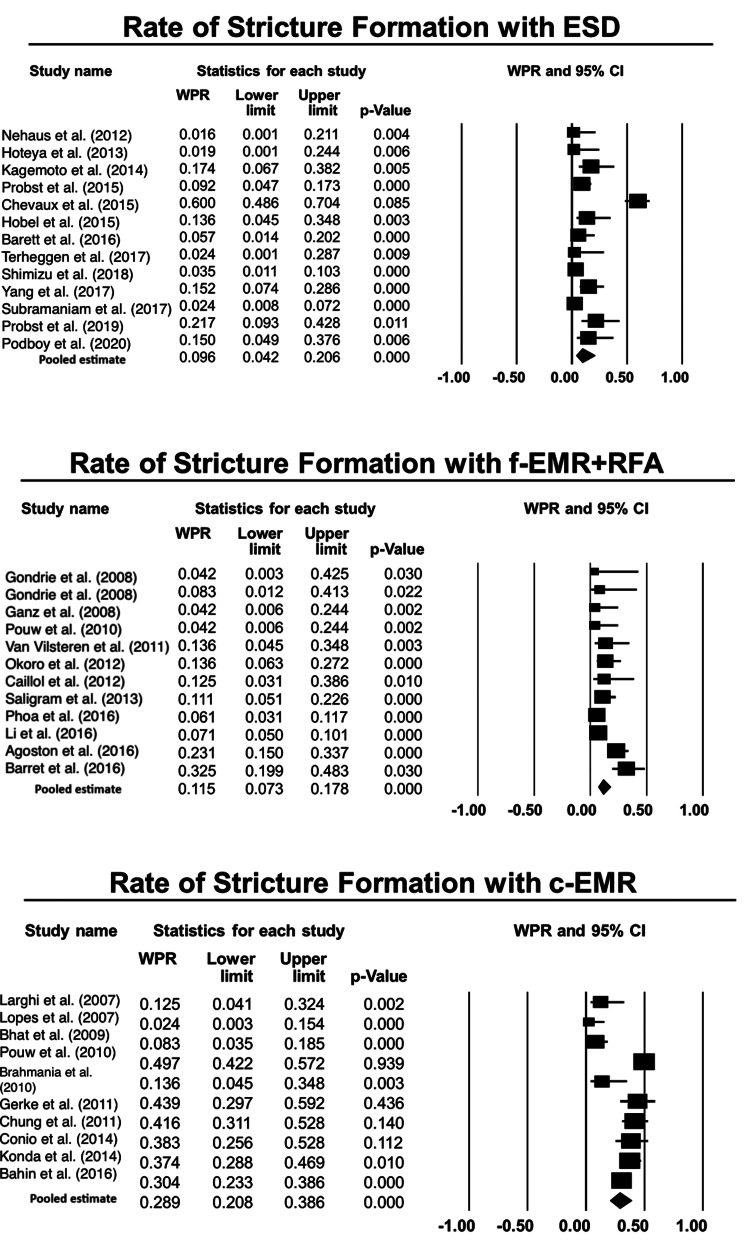
Forest plots for the included studies evaluating the rate of stricture formation associated with each technique, viz., EMR, ESD, and RFA. ESD: endoscopic submucosal dissection; f-EMR+RFA: focal endoscopic mucosal resection + radiofrequency ablation; c-EMR: complete endoscopic mucosal resection Weighted pooled rates (WPR) along with 95% confidence intervals (CIs).

Risk of stricture formation during follow-up: There was no difference in risk of stricture development based on proportional difference while comparing ESD with f-EMR+RFA was 2% (-1%, 5%), p=0.41 (Figure 4) [1,4,7,13-15,17-27,29-36,38-46]. The proportional difference while comparing ESD with c-EMR was 19% (15%, 23%), p<0.001 in favor of ESD. Likewise, f-EMR+RFA was also associated with significantly lower risk for stricture development when compared with c-EMR, the proportional difference being 17% (13%, 21%), p<0.001, as shown in Table 4.

**Figure 5:**
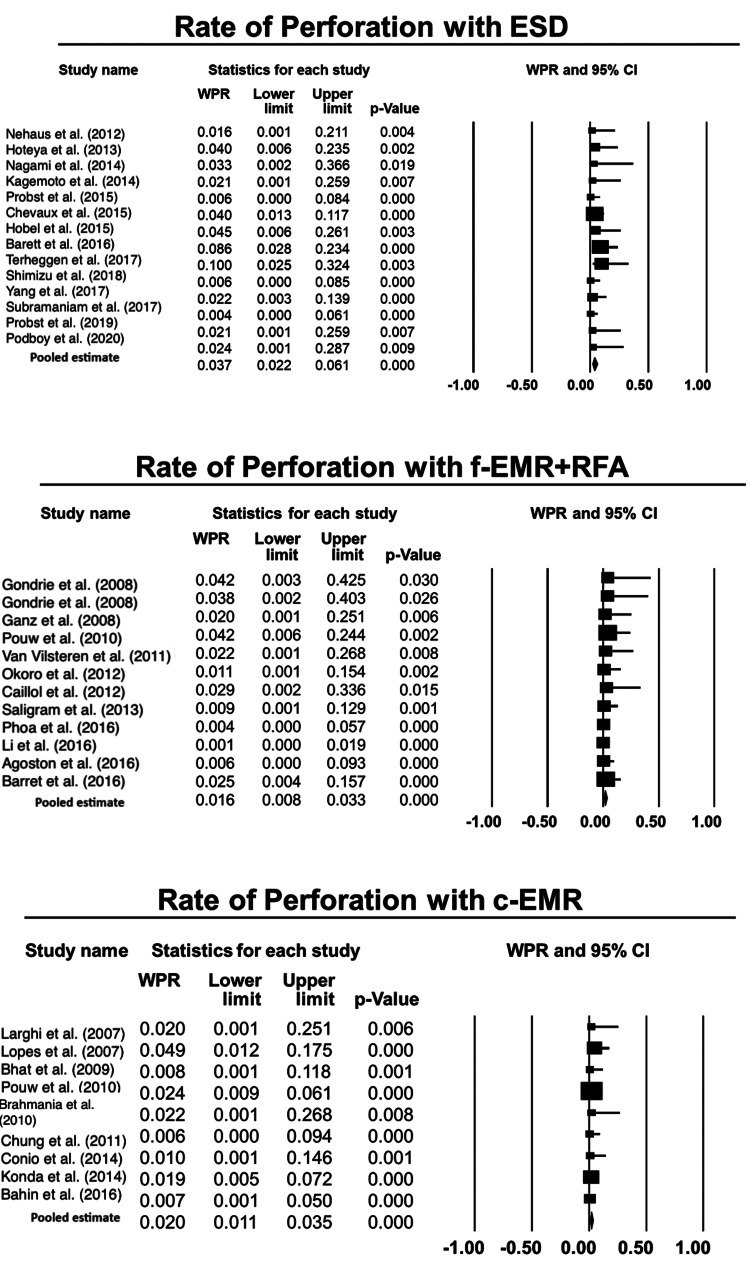
Forest plots for the included studies evaluating the rate of perforation associated with each technique, viz., EMR, ESD, and RFA. ESD: endoscopic submucosal dissection; f-EMR+RFA: focal endoscopic mucosal resection + radiofrequency ablation; c-EMR: complete endoscopic mucosal resection Weighted pooled rates (WPR) along with 95% confidence intervals (CIs).

Risk of perforation: f-EMR+RFA was associated with a lower risk of perforation compared to ESD, the proportional difference being 2% (0.5%, 3%), p=0.01 (Figure 5). There was no difference in the risk of perforation when ESD was compared with c-EMR 1% (-0.1%, 3%), p=0.07. Finally, there was no difference in the risk of perforation when f-EMR+RFA was compared with c-EMR 0.4% (-0.1%, 1%), p=0.5, as shown in Table 4.

Discussion

EMR has been the treatment modality of choice for resectioning of visible neoplastic lesions (HGD and EAC) in BE, as it is technically easy, safe, and effective. ESD is being increasingly utilized for the management of superficial neoplastic lesions in the GI tract, and this concept has been extended to BE-related neoplasia. ESD has the advantage of providing en bloc and R0 resections as compared to EMR, which is usually performed in a piecemeal fashion for lesions larger than 1.5 cm. Piecemeal resection could hypothetically limit histological diagnosis with risk of missing advanced neoplastic lesions [48]. Incidence of recurrence has been reported to be higher with piecemeal resection as compared to en bloc resections [49].

On the other hand, ESD is a technically advanced procedure requiring lengthy procedure times and has had reported adverse event rates of 4-67% of patients [35,41]. The only comparative study evaluating the success of ESD in comparison to f-EMR+RFA did not show any difference in the recurrence of neoplasia [7]. This study may have been underpowered for such comparisons, thus justifying the need for this indirect comparison meta-analysis to evaluate the efficacy and safety of three different endoscopic approaches, viz., f-EMR+RFA, c-EMR, and ESD for management of BE-related neoplasia.

We found that all three endoscopic modalities have excellent efficacy in the management of BE-related neoplasia. ESD showed en bloc resection rates of 89.5% and R0 resection rates of 72% with a recurrence rate of 9%. Likewise, f-EMR+RFA and c-EMR showed complete eradication rates of BE-related neoplasia of 93% and 94%, respectively. The rate of recurrence of BE-related neoplasia was 5% and 7.2% for f-EMR+RFA and c-EMR, respectively. EMR studies did not report en bloc and R0 resection rates, as piecemeal resections are more common with EMR. However, complete eradication of neoplasia was not reported in ESD studies, which were focused more on R0 and complete resection rates. Terheggen et al. reported the only comparative trial for ESD and EMR and did not note any difference in complete eradication of neoplasia between the two modalities [7]. For an indirect comparative analysis of efficacy, we chose the outcome of recurrence because it was the only uniform outcome reported by all studies. There was no difference in rates of recurrence with any of the three modalities.

For safety analysis, we focused our attention on the following three most common and uniformly reported adverse events: bleeding, stricture formation, and perforation. ESD and f-EMR+RFA had a lower risk of bleeding compared to c-EMR, which may be attributed to the radical endoscopic resection technique associated with c-EMR, which may have resulted in a 6% rate of bleeding. The risk of stricture formation during follow-up was similar between ESD and f-EMR+RFA, while both modalities were associated with significantly lower rates of stricture formation when compared to c-EMR. These results contradict the notion that ESD may be associated with very high rates of stricture formation [50]. Rather, wide-field endoscopic resections, particularly when including more than three-fourths of the esophageal circumference, have been associated with higher risk of stricture development [51-53]. Therefore, the risk of stricture formation was higher in the more radical endoscopic resections associated with c-EMR. f-EMR+RFA was associated with a lower risk of perforation compared to ESD, while both ESD and c-EMR had similar risks of perforation.

To the best of our knowledge, this is the first systematic review and pooled analysis to not only evaluate the cumulative efficacy of ESD, f-EMR+RFA, and c-EMR but also to provide an indirect comparative analysis and add substantially to the existing literature on the efficacy and safety of these endoscopic modalities for management of BE-related neoplasia. We conducted a comprehensive literature search, including all relevant studies to derive a meaningful comparison, and excluded small case series with less than 10 patients to minimize bias. For studies on c-EMR, we limited our inclusion criteria to those using c-EMR as a primary method to achieve complete eradication of BE-related neoplasia. For studies on f-EMR+RFA, we only included those with at least 70% of patients receiving f-EMR. We also limited our inclusion criteria to studies with at least one year of follow-up to provide a better assessment of the recurrence of neoplasia. Our analyses may be weakened by the limitations inherent to meta-analysis and those of the included studies. Only two RCTs were included, and the rest were observational studies. This indirect comparison analysis should be considered as exploratory only and may provide a platform for comparative trials for these endoscopic modalities in the future. Comparative outcomes in our study should be interpreted with caution because the assumption of independent random samples may not be satisfied due to the heterogeneity of study design, patient population, and reporting of outcomes. Further, we could not compare the complete eradication of neoplasia and the time required for procedures, as such data were not uniformly provided. We could not evaluate the effects on recurrence or adverse events based on patient demographics and lesion size. Evaluation of such variables would require an individual patient meta-analysis, and unfortunately, such data were not provided in a combinable manner by all studies. Among comparative variables, significant heterogeneity was encountered in stricture formation with all endoscopic modalities. Such heterogeneity may be explained by evolving techniques over the years, disparity in technical expertise, and treatment protocols. Such disparities would be overcome by conducting well-designed comparative trials.

One of the limitations of the study, as acknowledged above, is that without granular data about lesion size and features, such as a degree of differentiation, the results of the comparative outcomes could be difficult to interpret. Current guidelines recommend EMR with RFA for smaller lesions and ESD for larger, more bulky lesions or early-stage T1b EAC lesions. Since lesion characteristics play a significant role in the choice of resection technique, not having this information is a significant limitation. Additionally, it would have been informative if the primary outcome of recurrence after complete eradication was stratified by baseline dysplasia grade, as the risk of recurrence is higher with EAC than HGD. However, due to the nature of this study, this information was not available and hence could have limited the results.

We believe that the recurrence can be higher if patients are followed up for a long time and that recurrence is falsely lower if patients have a shorter follow-up. However, due to the nature of this study, we have reported the risk of recurrence as a total percentage as opposed to the annual rate of recurrence, which limited our results, as long-term follow-up data is not available.

Based on this pooled analysis, f-EMR+RFA is the preferred endoscopic modality for the management of BE-related neoplasia as it was associated with similar recurrence risk with a better safety profile compared to ESD in terms of perforation risk. The high rates of R0 resection with ESD did not translate into lower recurrence risk. Both f-EMR+RFA and ESD were better than c-EMR in terms of risk of bleeding and stricture development during follow-up. RCTs comparing the efficacy and safety of ESD and f-EMR are required.

## Conclusions

Based on this pooled analysis, f-EMR+RFA is the preferred endoscopic modality for the management of BE-related neoplasia as it was associated with similar recurrence risk with a better safety profile compared to ESD in terms of perforation risk. The high rates of R0 resection with ESD did not translate into lower recurrence risk. Both f-EMR+RFA and ESD were better than c-EMR in terms of risk of bleeding and stricture development during follow-up. RCTs comparing the efficacy and safety of ESD and f-EMR are required.
